# Extramural Venous Invasion as Prognostic Factor of Recurrence in Stage 1 and 2 Colon Cancer

**DOI:** 10.1155/2017/1598670

**Published:** 2017-11-28

**Authors:** E. E. van Eeghen, M. J. Flens, M. M. R. Mulder, R. J. L. F. Loffeld

**Affiliations:** ^1^Department of Internal Medicine, Zaans Medisch Centrum, Zaandam, Netherlands; ^2^Department of Pathology, Zaans Medisch Centrum, Zaandam, Netherlands

## Abstract

**Aim:**

Extramural venous invasion (EMVI) is a prognostic indicator in patients with colorectal cancer. However, its additional value in patients with stage 1 and 2 colorectal cancer is uncertain. In the present study, the incidence of EMVI and the hazard ratio for recurrence in patients with stage 1 and 2 colon cancer were studied.

**Methods:**

184 patients treated for stage 1 and 2 colon cancer were included with a follow-up of at least 5 years. Chart review was performed and EMVI was assessed by two separate pathologists. EMVI was scored with additional caldesmon staining on the resection specimen. Primary outcomes were recurrence-free survival (RFS) measured through the Cox regression analysis and prevalence of EMVI.

**Results:**

There were 10 cases of EMVI and 3 cases of intramural venous invasion (IMVI) all occurring in patients with stage 2 disease corresponding to a prevalence of 9%. Thirty-one percent of the patients with venous invasion experienced recurrence versus 14% in patients without, corresponding with a hazard ratio of 2.39 (*p* = 0.11).

**Conclusion:**

The present study demonstrates a trend towards an increased risk of recurrence in patients with stage 2 colon cancer with venous invasion. This warrants consideration of adjuvant chemotherapy despite the lack of lymph node metastases.

## 1. Introduction

Colon cancer is one of the most occurring malignancies in the Western world. Curative treatment is largely dictated by the TNM stage. A number of studies have demonstrated the benefit of adjuvant chemotherapy in stage 3 colon cancer [1, 2]. This benefit is less clear in patients with stage 2 disease, and as such, clinicians have tried to confine treatment to patients with tumours with characteristics that present a high risk of recurrence [3–6].

A previously underappreciated predictor of recurrence is extramural venous invasion (EMVI). Previous studies report hazard ratios of recurrence between 1.5 and 2.7 for patients with EMVI [7–10]. However, the inclusion criteria between studies differ, as does the reported prevalence of VI ranging from 23% to 28% in recent studies. The incidence of EMVI is correlated with disease stage being as low as 3% in stage 1 patients, up to 53% in patients with stage 4 disease in the study by Gibson et al. [7]. Discrepancies in prevalence might also be the result of different staining techniques used in different studies, with some studies providing only haematoxylin/eosin (HE) staining. This method might lack sensitivity in detecting EMVI [11–13]. Roxburgh et al. conducted a study comparing 2 cohorts that had been analyzed through HE with or without additional elastic staining. The incidence of VI was 18% versus 58% in favour of elastica staining while 3-year survival remained similar in both VI-positive groups (77% and 75%) [14]. The present study is done in order to establish the prevalence of VI in stage 1 and 2 colon cancer using a caldesmon staining technique to increase sensitivity for intra- or extramural invasion. Secondly, the hazard ratio for recurrence of tumour in patients with stage 1 and 2 cancer with and without VI was assessed.

## 2. Methods

All patients with stage 1 and 2 colon cancer treated at the Zaans Medisch Centrum, the regional hospital in Zaanstreek region, in the Netherlands, between 2002 and 2008 were included. This was part of a much larger study on disease-free and overall survival in patients with colorectal cancer. The analysis was done on January 1, 2014, providing at least 5 years of follow-up for all patients. An extensive chart review was conducted for all patients in order to retrieve tumour and patient characteristics. Except for venous invasion, all tumour characteristics are reported as described in the original pathology report. The Charlson age-comorbidity scale was used to assess patient comorbidity [15, 16].

Postoperatively, the resection specimens were routinely fixed in formalin and embedded in paraffin wax. The routine 3 *μ*m sections were stained with the standard haematoxylin/eosin stain. At the time of the final analysis, the patient was scored according to the TNM classification. For the present study, a tissue block with representative tumour was selected from the archive for immunohistochemical staining with caldesmon on the BOND III full automatic Leica stainer. The formalin fixed, paraffin wax-embedded sections were dewaxed. To enhance immunostaining, these sections were subjected to an epitope retrieval solution (high pH 9.0) for 20 minutes at 100°C. Endogenous peroxidase and nonspecific binding were blocked before addition of the primary antibody. The slides were stained with the monoclonal antibody caldesmon from Dako (Glostrup, Denmark), clone h-CD, 1 : 100.

All sections were stained with the standardized 3,3-diaminobenzidine tetrahydrochloride (DAB) conjugate kit from Leica and counterstained with haematoxylin.

**Figure 1:**
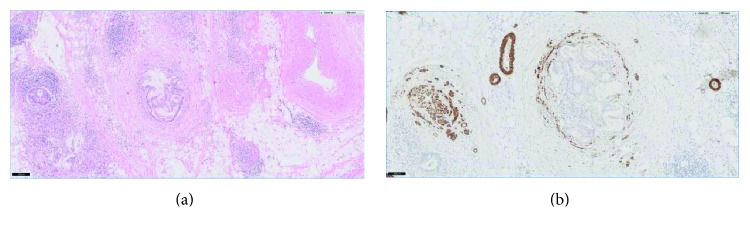
Overview of caldesmon staining of the venous vessel wall. (a) Haematoxylin and eosin staining shows a low-power view with a nest of invasive adenocarcinoma into the pericolic adipose tissue. (b) The caldesmon staining highlights the venous vessel wall around the tumour nest. The additional staining is an invaluable aid in the diagnosis of extramural venous invasion.

The presence of tumour cells within venous structures beyond the bowel wall was assessed on all haematoxylin and eosin-stained sections of the tumour and with the additional use of caldesmon staining in representative and most suspected area of the tumour. The caldesmon staining is superior to the standard haematoxylin and eosin staining. Highlighting the vessels by caldesmon staining significantly increases the observed incidence of vascular invasion in colorectal cancer compared with haematoxylin and eosin alone [17–19] (Figure 1).

Two experienced pathologists, MF and MM, scored the immunostaining results. They were blinded for patient outcomes. After independent assessment, cases coded as diagnostically discordant were discussed in a pathology panel discussion for consensus.

Log-rank and multivariate Cox regression analyses were used to determine the hazard ratio of recurrence for patients with EMVI. In addition, a T4 tumour, lymph node yield, and poor histological differentiation were analyzed with respect to recurrence. Statistical analyses were performed using IBM SPSS 20.0. A *p* value below 0.05 was considered statistically significant.

## 3. Results

A total of 192 patients were treated for stage 1 and 2 colon cancer. From eight patients, the original HE slides and/or the resection specimen were not available for reassessment; therefore, these patients were excluded (Table 1).  Table 2 shows the demographics and the localization of the tumour.  Table 3 shows the characteristics of recurrence. Also, the Charlson index, differentiation grade, and the number of lymph nodes are noted.

**Figure 2:**
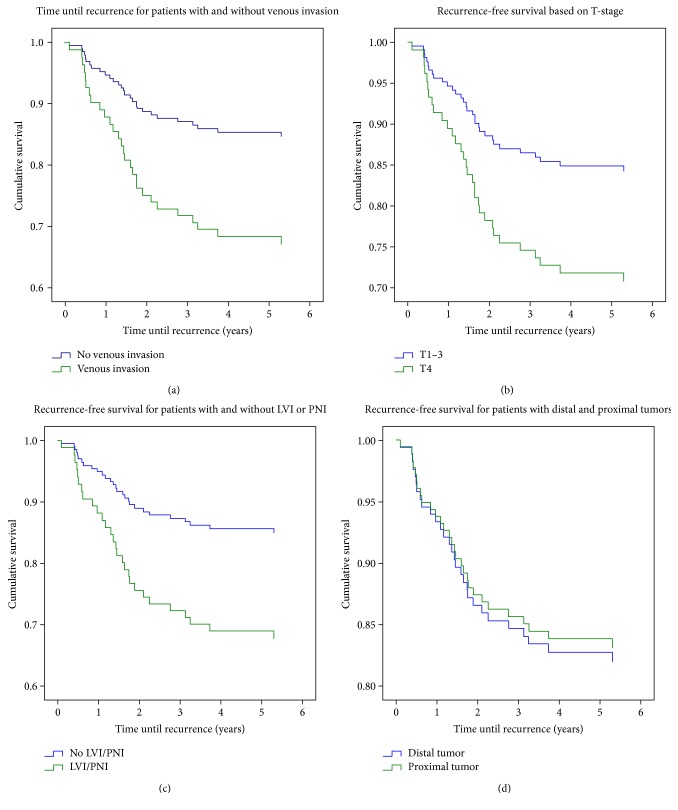
(a–d) Kaplan-Meyer plots reporting the association of tumour characteristics and recurrence-free survival. Patients were censored if death occurred before recurrence. (a) IM ± EMVI, (b) T4 tumour, (c) LVI/PNI, and (d) distal versus proximal tumours. LVI/PNI: lymphovascular or perineural invasion.

In the remaining 184 patients, 10 (5.4%) cases of EMVI and 3 (1.6%) cases of IMVI were observed. EMVI was only diagnosed in patients with stage 2 disease. Three patients in the EMVI group experienced recurrent disease (30%), one in the IMVI group (33%), and 24 in the control group (patients without VI) (14%). Univariate Cox regression analyses yielded a hazard ratio of 2.39 for recurrence in the group with EMVI (*p* = 0.107) (Figure 2(a)). The Cox regression analysis for other tumour characteristics also known to cause an increased risk of recurrence-free survival showed no significant outcomes. Hazard ratio for the recurrence for T4 tumours was 2.02 (Figure 2(b)). Every (negative) lymph node examined yielded a 4.0% reduction in risk of recurrence. Patients with LVI/PNI had a hazard ratio for recurrence of 2.21 (Figure 2(c)). Proximal tumours were associated with a hazard ratio for recurrence of 0.93 (Figure 2(d)). No analysis was performed for the differentiation grade as none of the 13 patients with poorly differentiated tumours experienced recurrence.

## 4. Discussion

This study adds to the evidence that venous invasion is a predictor of recurrence in colon cancer. This study is unique since only patients with stage 1 and 2 colon cancer are included. Most studies presented in the literature report on disease-free survival and recurrence-free survival while only a minority of the studied patients actually have a follow-up after treatment of five years. A trend was found with respect to recurrence in patients with VI in stage 2 colon cancer. A possible drawback could be the relatively low number of patients. The recurrence rate was an alarming 31% in patients with VI, compared to 10–20% in all patients with stage 2 disease [3, 4]. The hazard ratio of recurrence of 2.39 was similar to that reported by other studies. Although in most studies, hazard ratios included patients with (locally) advanced disease. Gibson et al. only found a significant increase of recurrence in patients with stage 3 disease [7]. The study by Baumhoer et al. found a marked increase in detection of VI with elastic staining but did not find an association between the presence of venous invasion and risk of recurrence in patients with stage 2 colon cancer [20]. Several other studies did find an association between (recurrence-free) survival and venous invasion in patients with stage 2 disease [8, 10, 21, 22]. Accurately determining the risk of recurrence is most important in patients with stage 2 disease as adjuvant chemotherapy is not usually indicated but can be added to improve prognosis in high-risk patients.

Routine haematoxylin/eosin staining is not sufficient to detect extramural vascular invasion.

More specific stains have to be used [17]. The caldesmon stain is by far the most accurate. After analysis by two GI-specialized pathologists, prevalence of venous invasion appeared to be rather low with 0% and 9% in patients with stage 1 and 2 disease, respectively. Detection rates of VI in patients with stage 2 disease range from 10 to 34% in previous studies [7, 8, 10, 17, 21, 23]. Because of the influence of EMVI on recurrence risk, the Royal College of Pathologists has added VI to the “core data items” and stipulates a minimum overall detection rate of VI of 30% as audit criterion recommending the use of elastica staining and the evaluation of at least 2 blocks, especially if the detection rates are not matched [24]. However, a Canadian survey shows that these detection rates are currently far from being met [19]. Secondly, with the introduction of colorectal cancer screening, the percentage of patients presenting with local disease increases; thus, the incidence of EMVI will decline [25]. Conversely, this increase in local disease further underlines the need for proper risk stratification of these patients.

In conclusion, this study adds to previous evidence demonstrating an increased risk of recurrence in patients with stage 2 colon cancer with venous invasion. In this study, thirty-one percent of these high-risk patients experienced recurrence. Sensitivity of diagnostic tests for venous invasion will influence its specificity as a prognostic marker. Prevalence of EMVI varies between studies and pathologists, and more precise guidelines with regard to the staining and number of evaluated blocks should be formulated. This is especially important for patients with stage 2 disease, as the high recurrence rate for EMVI-positive tumours warrants consideration for adjuvant chemotherapy despite the absence of lymph node metastases. One should also take into account the presence of additional risk factors for recurrence (e.g., a T4 tumour).

## Figures and Tables

**Table 1:** Characteristics of missing patients.

Number of patients	8	
Gender		
Male	5	62.5%
Female	3	37.5%
Age^∗^	62.5	(52.3–73.1)
Charlson age index^∗^	3	(2–4.75)
T-stage		
1	1	12.5%
2	0	0%
3	5	62.5%
4	2	25%
Differentiation		
Poor	7	100%
Well	0	0%
Adjuvant treatment	7	87.5%
LVI/PNI	1	12.5%
Number of examined lymph nodes^∗^	7.5	(5–15.25)
Tumour location		
Distal	5	62.5%
Proximal	1	12.5%
Synchronous	2	25%
Recurrence	3	37.5%
Overall survival^∗^	4.89	(3.25–8.34)
Recurrence-free survival^∗^	4.82	(1.76–8.14)
Cause of death		
Alive	4	50%
Tumour	3	37.5%
Complication of treatment	0	0%
Other	0	0%
Unknown	1	12.5%

EMVI: extramural invasion; IMVI: intramural invasion; LVI/PNI: lymphovascular invasion/perineural invasion. ^∗^Median and interquartile range.

**Table 2:** Patient characteristics.

	EMVI		IMVI		No EMVI	
Number of patients	10		3		171	
Gender						
Male	5	50%	0	0%	94	55%
Female	5	50%	3	100%	77	45%
Age^∗^	78	(56–85)	70		73	(66–80)
Charlson age index^∗^	5.5	(2.75–7.25)	5		5	(3–6)
T-stage						
1	0	0%	0	0%	10	6%
2	0	0%	0	0%	33	19%
3	9	90%	3	100%	106	62%
4	1	10%	0	0%	22	13%
Differentiation						
Poor	0	0%	0	0%	13	8%
Well	10	100%	3	100%	151	92%
Adjuvant treatment	0	0%	0	0%	8	5%
LVI/PNI	2	80%	0	0%	14	8%
Number of examined lymph nodes^∗^	8.5	(5.5–15.25)	19		13	(8–18)
Tumour location						
Distal	6	60%	0	0%	77	45%
Proximal	4	40%	3	100%	86	50%
Synchronous	0	0%	0	0%	8	5%
Recurrence	3	30%	1	33%	24	14%
Overall survival^∗^	5.96	1.21–9.24	5.58		6.75	5.09–8.59
Cause of death						
Alive	4	40%	0	0%	108	63%
Tumour	2	20%	1	33%	15	9%
Complication of treatment	0	0%	0	0%	6	4%
Other	3	30%	2	67%	31	18%
Unknown	1	10%	0	0%	11	6%

^∗^Median and interquartile range. No interquartile ranges were reported for the IMVI group due to the small number of patients. EMVI: extramural invasion; IMVI: intramural invasion; LVI/PNI: lymphovascular invasion/perineural invasion.

**Table 3:** Characteristics associated with recurrence. Tumour location is divided in tumour distal and proximal to the flexura lienalis. Hazard ratios are determined through univariate Cox regression analysis.

	Cox hazard ratio	95% confidence interval
EM ± IMVI	2.39	0.83–6.89
T4	2.02	0.87–4.69
Differentiation	No events in patients with poor differentiation	
LVI/PNI	2.21	0.85–5.75
Number of examined lymph nodes^∗^	0.96	0.92–1.01
Tumour location (distal is reference)	0.93	0.45–1.9

^∗^Hazard ratios are reported for each additional lymph node examined. EM ± IMVI: extramural and intramural venous invasion, LVI/PNI: lymphovascular/perineural invasion.

## References

[1] Andre T., Boni C., Mounedji-Boudiaf L. (2004). Oxaliplatin, fluorouracil, and leucovorin as adjuvant treatment for colon cancer. *The New England Journal of Medicine*.

[2] Kuebler J. P., Wieand H. S., O'Connell M. J. (2007). Oxaliplatin combined with weekly bolus fluorouracil and leucovorin as surgical adjuvant chemotherapy for stage II and III colon cancer: results from NSABP C-07. *Journal of Clinical Oncology*.

[3] Schippinger W., Samonigg H., Schaberl-Moser R. (2007). A prospective randomised phase III trial of adjuvant chemotherapy with 5-fluorouracil and leucovorin in patients with stage II colon cancer. *British Journal of Cancer*.

[4] Quah H. M., Chou J. F., Gonen M. (2008). Identification of patients with high-risk stage II colon cancer for adjuvant therapy. *Diseases of the Colon & Rectum*.

[5] Yamaguchi K., Ogata Y., Akagi Y., Shirouzu K. (2013). Identification of high‑risk factors as indicators for adjuvant therapy in stage II colon cancer patients treated at a single institution. *Oncology Letters*.

[6] McKenzie S., Nelson R., Mailey B. (2011). Adjuvant chemotherapy improves survival in patients with American Joint Committee on Cancer stage II colon cancer. *Cancer*.

[7] Gibson K. M., Chan C., Chapuis P. H., Dent O. F., Bokey L. (2014). Mural and extramural venous invasion and prognosis in colorectal cancer. *Diseases of the Colon & Rectum*.

[8] Betge J., Pollheimer M. J., Lindtner R. A. (2012). Intramural and extramural vascular invasion in colorectal cancer: prognostic significance and quality of pathology reporting. *Cancer*.

[9] Parnaby C. N., Scott N. W., Ramsay G. (2015). Prognostic value of lymph node ratio and extramural vascular invasion on survival for patients undergoing curative colon cancer resection. *British Journal of Cancer*.

[10] Petersen V. C., Baxter K. J., Love S. B., Shepherd N. A. (2002). Identification of objective pathological prognostic determinants and models of prognosis in Dukes’ B colon cancer. *Gut*.

[11] Messenger D. E., Driman D. K., Kirsch R. (2012). Developments in the assessment of venous invasion in colorectal cancer: implications for future practice and patient outcome. *Human Pathology*.

[12] Kirsch R., Messenger D. E., Riddell R. H. (2013). Venous invasion in colorectal cancer: impact of an elastin stain on detection and interobserver agreement among gastrointestinal and nongastrointestinal pathologists. *The American Journal of Surgical Pathology*.

[13] Howlett C. J., Tweedie E. J., Driman D. K. (2009). Use of an elastic stain to show venous invasion in colorectal carcinoma: a simple technique for detection of an important prognostic factor. *Journal of Clinical Pathology*.

[14] Roxburgh C. S. D., McMillan D. C., Anderson J. H., McKee R. F., Horgan P. G., Foulis A. K. (2010). Elastica staining for venous invasion results in superior prediction of cancer-specific survival in colorectal cancer. *Annals of Surgery*.

[15] Marventano S., Grosso G., Mistretta A. (2014). Evaluation of four comorbidity indices and Charlson comorbidity index adjustment for colorectal cancer patients. *International Journal of Colorectal Disease*.

[16] Ouellette J., Small D., Termuhlen P. (2004). Evaluation of Charlson-age comorbidity index as predictor of morbidity and mortality in patients with colorectal carcinoma. *Journal of Gastrointestinal Surgery*.

[17] Kingston E. F., Goulding H., Bateman A. C. (2007). Vascular invasion is underrecognized in colorectal cancer using conventional hematoxylin and eosin staining. *Diseases of the Colon & Rectum*.

[18] Stewart C. J. R., Hillery S., Platell C. (2012). Caldesmon is useful in demonstrating extramural venous invasion in colorectal carcinomas showing mucinous differentiation. *Pathology*.

[19] Dawson H., Kirsch R., Driman D. K., Messenger D. E., Assarzadegan N., Riddell R. H. (2015). Optimizing the detection of venous invasion in colorectal cancer: the Ontario, Canada, experience and beyond. *Frontiers in Oncology*.

[20] Baumhoer D., Thiesler T., Maurer C. A., Huber A., Cathomas G. (2010). Impact of using elastic stains for detection of venous invasion in the prognosis of patients with lymph node negative colorectal cancer. *International Journal of Colorectal Disease*.

[21] Morris E. J. A., Maughan N. J., Forman D., Quirke P. (2007). Who to treat with adjuvant therapy in Dukes B/stage II colorectal cancer? The need for high quality pathology. *Gut*.

[22] McClelland D., Murray G. I. (2015). A comprehensive study of extramural venous invasion in colorectal cancer. *PLoS One*.

[23] Talbot I. C., Ritchie S., Leighton M., Hughes A. O., Bussey H. J. R., Morson B. C. (1981). Invasion of veins by carcinoma of rectum: method of detection, histological features and significance. *Histopathology*.

[24] Loughrey M. B., Quirke P., Shepherd N. A. (2014). Dataset for colorectal cancer histopathology reports.

[25] Mansouri D., McMillan D. C., Crighton E. M., Horgan P. G. (2013). Screening for colorectal cancer: what is the impact on the determinants of outcome?. *Critical Reviews in Oncology/Hematology*.

